# Regulation of PD-L1: Emerging Routes for Targeting Tumor Immune Evasion

**DOI:** 10.3389/fphar.2018.00536

**Published:** 2018-05-22

**Authors:** Yiting Wang, Huanbin Wang, Han Yao, Chushu Li, Jing-Yuan Fang, Jie Xu

**Affiliations:** MOH Key Laboratory of Gastroenterology and Hepatology, State Key Laboratory for Oncogenes and Related Genes, Renji Hospital, School of Medicine, Shanghai Jiao Tong University, Shanghai, China

**Keywords:** PD-L1, immunotherapy, gene expression, post-translational modification, small molecular inhibitors

## Abstract

Immune checkpoint blockade therapies (ICBTs) targeting programmed cell death 1 (PD-1) and its ligand programmed death ligand-1 (PD-L1/B7-H1/CD274) have exhibited momentous clinical benefits and durable responses in multiple tumor types. However, primary resistance is found in considerable number of cancer patients, and most responders eventually develop acquired resistance to ICBT. To tackle these challenges, it is essential to understand how PD-L1 is controlled by cancer cells to evade immune surveillance. Recent research has shed new light into the mechanisms of PD-L1 regulation at genetic, epigenetic, transcriptional, translational, and posttranslational levels. In this work, we systematically discuss the mechanisms that control the gene amplification, epigenetic alteration, transcription, subcellular transportation and posttranscriptional modification of PD-L1 in cancer cells. We further categorize posttranscriptional PD-L1 regulations by the molecular modification of PD-L1, including glycosylation, phosphorylation, ubiquitination, deubiquitination, and lysosomal degradation. These findings may provide new routes for targeting tumor immune escape and catalyze the development of small molecular inhibitors of PD-L1 in addition to existing antibody drugs.

## Introduction

Over the past decades, a novel therapy that utilizes human immune system to treat cancer is increasingly popular, which is known as cancer immunotherapy (Yang, 2015). The immunosuppressive microenvironment of tumor is one of the six distinct biological properties that enable tumor growth and metastasis (Hanahan and Weinberg, 2011). Human tumors typically harbor genomic instability, which induce somatic mutations (Hanahan and Weinberg, 2011). Accumulation of mutations may facilitate tumor growth and metastasis, while some non-synonymous mutations, leading to replacement of amino acid residual, create new T cell epitopes (neoepitopes), offering opportunities for immune system to recognize and eliminate cancer cells (Matsushita et al., 2012; Rooney et al., 2015). It has been reported that the number of non-synonymous mutations, defined as mutational load, is closely related with the efficacy of immunotherapy (Danilova et al., 2016). However, cancer cells collaborate with immune cells to dodge the immune destruction, and the anti-cancer pathway is intervened in this microenvironment (Blank et al., 2016; Sukari et al., 2016). The depressed immunology of T cells, if appropriately empowered, may be an efficient and powerful weapon against cancer. Specifically, active vaccination, adoptive cell transfer therapy and immune checkpoint blockade are the three major approaches that could turn on T cell-based anti-cancer immune reaction. In recent years, immune checkpoint blockade therapy (ICBT) has exhibited momentous clinical benefits, placing tumor immunotherapy under the spotlight (Sukari et al., 2016). PD-L1, a type I transmembrane protein with an extracellular N-terminal domain, inhibits the immune response through interaction with receptor PD-1 expressed on T cells (Horita et al., 2017). Under physiological conditions, PD-L1 is expressed in a wide range of cell types and tissues and shown to be overexpressed with immune activation, such as inflammations (Ritprajak and Azuma, 2015). The PD-L1/PD-1 axis maintains the balance between tolerance and autoimmunity and thus deficiency or excess function of it can lead to a variety of disease. Many auto-immune diseases have been found to be associated with PD-L1/PD-1 disruption including arthritis and lupus (Zamani et al., 2016). PD-L1 expression has been found positive in 5–40% tumor cells (Xie et al., 2016; Xiang et al., 2018), helping them to dodge the immune elimination through interaction of PD-L1 on the surface of cancer cells with PD-1 on T cells (Topalian et al., 2015). Thus, blockade of PD-L1/PD-1 axis assists the recognition and elimination of cancer cells. PD-L1 expression on tumor cells has been reasonably detected as a biomarker of ICBT (Ma et al., 2016). Further investigation revealed that the inducible but not continuous expression of PD-L1 is associated with activated CD8+ T cells in hepatocellular carcinoma (Xie et al., 2016), although the expression of PD-L1 is not independently prognostic (Wang X. et al., 2016; Xie et al., 2016).

The binding of immune checkpoint inhibitors and optimal targets is the core idea of ICBT. By inhibiting the immune-suppressive pathways, ICBT allows the clearance of cancer cells by the immune system (Topalian et al., 2015). Several immune checkpoints are discovered to be optimal targets for immune blockade, including the cytotoxic T-lymphocyte-associated protein 4 (CTLA-4) and programmed cell-death protein 1 (PD-1)/programmed cell-death 1 ligand 1 (PD-L1) pathways. Drugs targeting these two pathways have nourished recently and many of them have been approved by FDA. Drugs that target PD-1 like Pembrolizumab (Keytruda) and Nivolumab (Opdivo) were approved in 2014. Some PD-L1 inhibitors were also approved including Atezolizumab (Tecentriq) (2016), Avelumab (Bavencio) (2017) and Durvalumab (Imfinzi) (2017). Ipilimumab (Yervoy) is a monoclonal antibody targeting CTLA-4 that gained approval in 2011. Information comes from the official website of United States Food and Drug Administration. Notably, inhibitors targeting PD-1 or PD-L1 have been found to be especially advantageous in the treatment of many kind of cancer, including non-small cell lung carcinoma (NSCLC) (Wang C. et al., 2016), renal cell carcinoma (RCC), bladder cancer, breast cancer (Hu et al., 2017), melanoma (Luke et al., 2017) and Hodgkin’s lymphoma (Allen and Gordon, 2016). The landscape of cancer therapy is evolving with deeper and wider acknowledgment of Immunotherapy with PD-1 or PD-L1 blockade (Pardoll, 2012).

Despite of the promising laboratory results and many positive clinical applications, there seems to be a discount on its overall clinical benefits due to intrinsic and/or acquired resistance to this therapy (Sharma et al., 2017). In certain cancer patients, the significant clinical response and enduring tumor retardation achieved by ICBT have improved patient progress-free survival (PFS) and overall survival (OS). However, the efficacy rate and profits of usage in general patients remain at a modest level, impeding the widespread application of ICBT (Pardoll, 2012). The tumor immunogenicity is a multi-level and delicately modulated process. Therefore, accumulation of mutations may lead to dysregulation of immunogenicity and create an immunosuppressive microenvironment, causing intrinsic resistance to ICBT (Zhao and Subramanian, 2017). Among them is the insufficiency of T cell infiltration (Spranger et al., 2016; Tang et al., 2016). On the other hand, after the significant retardation and durable response of tumor when initially treated with anti-PD-1 therapy, relapses in the long term were observed even after continuous therapy (Zaretsky et al., 2016). The acquired resistance to ICBT in melanoma was reported to be associated to antigen presentation deficiency, in which the interferon signal pathway was involved (Zaretsky et al., 2016). Alternative checkpoints were discovered to be adaptively upregulated after PD-L1 targeting treatment (Koyama et al., 2016). Moreover, PD-L1 upregulation after chemotherapy and nivolumab treatment was reported as a potential cause of acquired resistance (Haratake et al., 2017). In these tumors, immune evasion involves PD-L1/PD-1 interaction, which is the reason why the therapy initially worked. But the aftereffect of increased PD-L1 may have partially restored PD-L1/PD-1 function by providing more PD-L1 sites that were not neutralized by injected antibodies. Nonetheless, not enough investigations have been done to clarify the adaptive upregulation of PD-L1. In this scenario, understanding the mechanisms of PD-L1 regulation in cancer cells would certainly benefit the development of more effective and durable ICBTs.

While the PD-1/PD-L1 pathway has been proven both theoretically and clinically a mature and efficient target for immunotherapy, it is of urgent need to develop more effective approaches to target PD-L1. Firstly, many disadvantages of PD-L1 targeted antibodies are unneglectable. The relatively large size of Mono-antibodies (MAbs) may prohibit its penetration into the complex tumor microenvironment, and thus limiting the therapeutic efficacy (Lee and Tannock, 2010). It is crucial to develop new drugs with smaller sizes and to improve the specificity of tumor PD-L1 targeting, even though existing drugs and research are flourishing (Tan et al., 2016).

Secondly, the primary and acquired resistance to ICBT in many tumors highlights a crucial requirement for developing alternative PD-L1/PD-1-targeting approaches. Several cancer mutations have been suggested to be the cause of PD-L1 suppression and therefore primary resistance to PD-L1 blockade drugs. Inactivation mutations of JAK1/2 is an example (Shin et al., 2017). Thirdly, As a protector of host tissue and regulator of inflammation, PD-1/PD-L1 is located not only on tumor cells but also on normal cells, including anti-tumor T cells and tumor associated macrophages (Tan et al., 2016; Horita et al., 2017). The blockage of physiological PD-1/PD-L1 functions inevitably brings about unfavored results- the depletion of cells which are meant to be activated and functioning. Lastly, the activation of oncogenic pathways, including RAS/RAF/MAPK and PI3K signaling, combined with the complexity of tumor microenvironment, may desensitize anti-tumor immunity (Zhao and Subramanian, 2018). The main components of tumor microenvironment, including infiltrated T cells (Tang et al., 2016), metabolites (will be further discussed) and oxidative stress (Maj et al., 2017), have been reported to be disruptors of anti-tumor immunity. Our understanding on the mechanisms of ICBT resistance and PD-L1 regulation remains rather limited, proposing an urgency to decode the multifaceted roles and complex control of PD-L1 in cancer.

The enthusiastic devotion from both clinical and biological investigators have brought the PD-1/PD-L1 biology into a new era in cancer research. Translational studies targeting the PD-1/PD-L1 pathway have boosted dramatically in recent years. Some progresses in the research of PD-L1 expression in cancer, especially at transcriptional and epigenetic levels, have been forged into a regulatory model for unified explanation (Chen et al., 2016). However, more recent findings that shed light into the multifaceted control of PD-L1 as a membranous protein has not been systematically discussed. In this review, we will summarize the exciting progresses in PD-L1 research in a more comprehensive manner, aiming to facilitate future basic and translational studies in the field of cancer immunotherapy.

## Genomic Alterations Drive PD-L1 Expression

**FIGURE 1:**
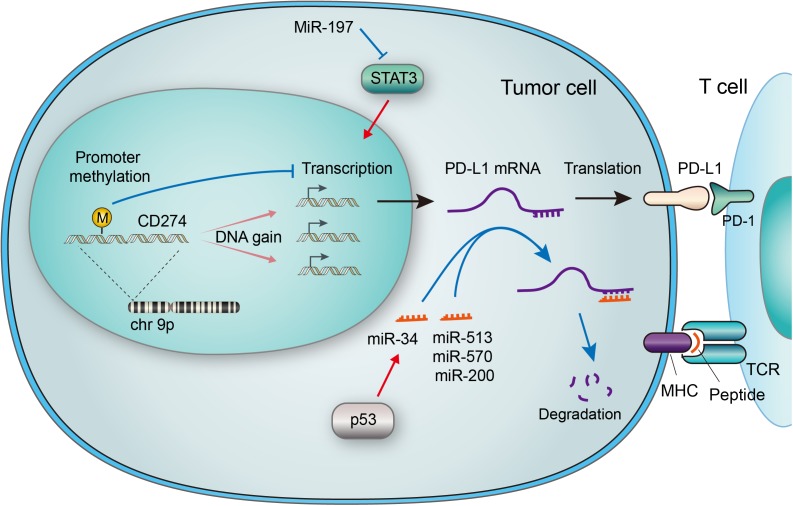
Genetic and epigenetic regulations of PD-L1 in tumor cells. The CD274 gene encoding PD-L1 is located on chromosome 9p, which is amplified in a subset of cancers. Increased gene copy number leads to upregulation of mRNA expression, while methylation of the gene promoter suppresses its transcription. Micro RNAs (miRNAs) may regulate PD-L1 expression by suppressing Stat3, which transactivates PD-L1. MiRNAs may also bind to the 3′ UTR of PD-L1 mRNA, leading to its degradation. The p53 tumor suppressor has been reported to downregulate PD-L1 through miR-34, a miRNA that binds to the 3′ UTR of PD-L1 mRNA. The PD-L1/PD-1 interaction and MHC-antigen/TCR interaction collaboratively define an inhibitory output of the immune checkpoint.

**FIGURE 2:**
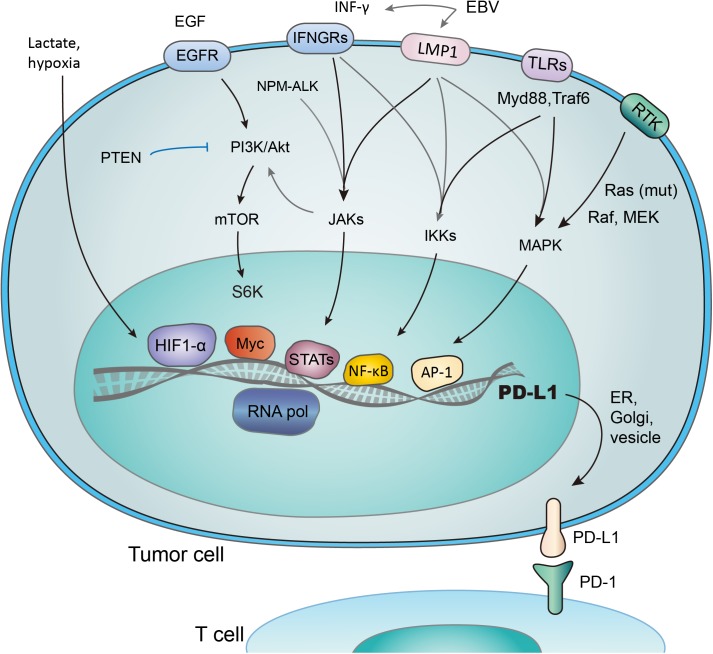
Transcriptional activation of PD-L1 in response to different signaling pathways. PD-L1 is transcribed in response to the activation of multiple signaling pathways, and transcription factors (TFs) such as HIF1-α, Myc, Stats, NF- κB, and AP-1 have been reported to bind and transactivate PD-L1. These TFs are controlled by the interconnected pathways involving EGF/PI3K/AKT/MTOR (suppressed by PTEN), RTK/Ras/Raf/MEK/ERK, IFN-γ/JAKs (also induced by mutant NPM-ALK gene and EBV-activated LMP1), TLRs/Myd88/Traf6/IKKs, and lactate-enriched microenvironment.

Enhanced PD-L1 expression was detected in a wide range of cancers but the prognostic and predictive value of it is controversial (Wang X. et al., 2016). It’s also a sign of efficacy of ICBT targeting PD-1/PD-L1 (Chen et al., 2016), as reported in B-cell lymphomas (Wang X. et al., 2016), breast cancer (Mittendorf et al., 2014), small-cell lung cancer (George et al., 2017) and pancreatic cancer (Wang et al., 2010). Given that many oncogenes are upregulated by gained copy number alterations (CNAs), efforts have been made to clarify the relationship between PD-L1 expression and CNA. As the main form of CNA, PD-L1 copy number amplification directly leads to PD-L1 mRNA upregulation. Tumors harboring PD-L1 amplification presents significantly higher load of mutation, comparing to non-amplified subjects (Budczies et al., 2016). Increased copy number of chromosome 9p24, predominant amplification of focal gene CD274 (which resides on chromosome 9p24.1, as shown in **Figure 1**), together with abundant PD-L1 expression were observed in a subset of small-cell lung cancer (SCLC) (George et al., 2017). The Janus kinase 2 (JAK2) amplification was documented to be simultaneously activated with 9p24.1 chromosome copy number amplification and upregulated PD-L1 expression in primary cancers (**Figure 2**), suggesting a possible transactivation between JAK2 and PD-L1 genes (Green et al., 2010; Ikeda et al., 2016; Clave et al., 2018). What’s more, PD-L1/PD-L2 alterations were defined as a feature of Classical Hodgkin lymphomas (cHLs). Specifically, amplification of 9p24.1 was reported to be associated with patients’ advanced stage disease and poor prognosis in cHL and in Epstein-Barr virus-associated gastric cancer (EBVaGC) (Roemer et al., 2016; Saito et al., 2017). These findings collectively suggest that CD274 gene amplification is a crucial factor that drives PD-L1 expression in cancer, and thus targeting PD-L1 at genetic level may be a rationalized strategy in PD-L1 positive tumors. Considering the rapid development of gene therapies, such prospect won’t be infeasible.

Structural variations may also be responsible for elevated transcription of PD-L1 (Kataoka et al., 2016). For example, truncation of its 3′UTR was reported to be associated with aberrant PD-L1 expression in multiple cancers (Kataoka et al., 2016).

## Epigenetic Regulation of PD-L1

Epigenetic regulation was revealed to be involved in PD-L1 expression in cancer cells. Micro RNAs (miRNAs), defined as 22–24 nucleotides non-coding single-stranded RNAs, have been implicated in the regulation of PD-L1 expression (Wang Q. et al., 2017). The binding of some miRNAs to the PD-L1 mRNA causes the latter one to degrade and thus PD-L1expression is suppressed. Specifically, the abundance of miR-513, miR-570, miR-34a, and miR-200 were reported to have an inverse correlation with PD-L1 expression (Chen, 2009; Chen et al., 2014; Wang et al., 2015), as described in **Figure 1**. Among them is miR-513 which inhibits PD-L1 protein translation by binding to 3′ untranslated regions (UTRs) of PD-L1 RNA as complement (Chen, 2009). Supportively, IFN-γ-induced PD-L1 expression was diminished by introducing miR-513 into Jurkat cells, while anti-miR-513 enhanced PD-L1 expression in cholangiocytes (Gong et al., 2009; Jardim et al., 2009). Similar function was found with miR-570. Research has shown that mutation of the PD-L1 3′ UTR which disrupts the association with miR-570, correlated with overexpression of PD-L1 (Wang et al., 2013). P53 was reported to regulate PD-L1 through miR-34 (Cortez et al., 2016). In the case of miR-200, the process of epithelial-to-mesenchymal transition (EMT) is found to be mediated by the regulation of PD-L1 expression by miR-200 (Chen et al., 2014). Moreover, MiR-197 was reported to repress STAT3, a regulator of PD-L1, to decrease PD-L1 expression (Fujita et al., 2015), as demonstrated in **Figure 1**. Other miRs reported to regulate PD-L1 includes miR-424 (Xu et al., 2016), miR-138 (Zhao et al., 2016), miR-17 (Audrito et al., 2017) and cluster miR-25-93-106b (Cioffi et al., 2017). Most recently, a mechanism that stabilizes PD-L1 mRNA was reported through modulation of the AU-rich element-binding protein tristetraprolin (TTP) (Coelho et al., 2017).

Recent studies have also focused on the promoter methylation of PD-L1 (mPD-L1), which was suggested to be a biomarker for prediction of response to PD-1/PD-L1 targeted ICBT. Significant inverse correlations between mPD-L1 and patient age was reported. The correlation between mPD-L1 and PD-L1 mRNA expression shares similar pattern, indicating a potential interaction between patient age and methylation of PD-L1 gene and that promoter methylation suppresses PD-L1 expression in colorectal cancer (CRC) (Goltz et al., 2017). Correlation between PD-L1 promoter methylation and clinical outcomes was also revealed in other cancers including NSCLC (Wrangle et al., 2013) and prostate cancer (Gevensleben et al., 2016). Moreover, in patients treated with PD-1/PD-L1 targeting drugs, enhanced mPD-L1 is associated with worse overall survival and recurrence-free survival. Epigenetic therapy has also been suggested to sensitize tumor response to PD-L1 targeting drugs (Wrangle et al., 2013). Interestingly, results proved no meaningful correlation between PD-L1 mRNA expression and patients’ outcome. (Goltz et al., 2017)

## Transcriptional Activation of PD-L1

Several transcriptional factors have been found to control PD-L1 transcriptional activation (**Figure 2**). As an example, PTEN represses PD-L1 transcription and expression in breast cancer cells, suggesting a new tumor suppressive function of PTEN. In addition, PD-L1 expression decreased after inhibition of phosphoinositide 3-kinase (PI3K) pathway using the AKT inhibitors, further emphasizing the role of PTEN and PI3K signaling in PD-L1 regulation (Mittendorf et al., 2014). Transcription activity, demonstrated by the level of PD-L1 mRNA expression, was promoted through JAK2/STAT1 pathway, as was shown in pancreatic cancer cells treated with anticancer agents (5-fluorouracil, gemcitabine, or paclitaxel) (Wang et al., 2010). Notably, when treated with chemotherapeutic drugs, the MAPK pathway was also reported to upregulate PD-L1 in cancer cells (Chen et al., 2016). While distinct signaling pathways share the ability to control PD-L1 expression by regulating its transcription, the exact mechanisms involved may vary considerably (Chen et al., 2016).

Hypoxia inducible factor 1α (HIF-1α) is a major cancer driver (Ortmann et al., 2014) and a potential therapeutic target (Brown and Wilson, 2004; Vaupel and Mayer, 2007; Wilson and Hay, 2011). The binding of HIF-1α to PD-L1 promoter, a hypoxia response element (HRE), stimulates the transcription of PD-L1 (Noman and Chouaib, 2014). Research has revealed the co-existence of HIF-1α overexpression, increased PD-L1 level, and repression of T-cell function (Noman et al., 2014; Pollizzi and Powell, 2014; Shehade et al., 2014). It was also reported that PD-L1 works predominantly in lactate-enriched tumor microenvironments (Feng et al., 2017). Meanwhile, T cell autophagy is induced in a microenvironment lack of amino acids tryptophan and arginine as well as glucose. In this nutrients-deprived situation, glucose metabolism shrinks while the lactate accumulates, creating an optimal environment for PD-1/PD-L1 interaction and resistance to cancer therapies consequently (Robainas et al., 2017). In other words, Lactate, as a major metabolite under hypoxia condition, may protect tumor cells from cytotoxic T-cell targeting. Accordingly, tumor cell metabolic reprograming was found to correlate with immune suppression (Feng et al., 2017). Taken together, it is suggested that hypoxic environments, which induce activation of HIF-1α and accumulation of lactate (Koukourakis et al., 2005; Marchiq and Pouyssegur, 2016; Ban et al., 2017), contribute to evasion of tumor cells from immune system. The transactivation of PD-L1 by HIF-1 represents a crucial step in the above-mentioned process, and may be a promising target to combat the immune suppression of tumor cells.

STAT3 is another important transcriptional factor that upregulates PD-L1 expression by binding to PD-L1 promoter. Mutations of oncogene chimeric nucleophosmin/anaplastic lymphoma kinase (ALK) have been found to upregulate PD-L1 expression, and this effect could be abolished by silencing STAT3 (Marzec et al., 2008). Furthermore, Latent membrane protein-1 (LMP1) of Epstein-Barr virus was found to increase both PD-L1 expression and STAT3 phosphorylation (p-STAT3) (Fang et al., 2014) (**Figure 2**). Consistently, the JAK3 inhibitor CP-690550 blocked the above process through suppressing p-STAT3 (Marzec et al., 2008). NF-κB, as a transcriptional factor mediating inflammation-associated tumorigenesis, has been reported to boost PD-L1 expression. However, the exact mechanisms remain unclear. NF-κB is required for LMP1-induced PD-L1 expression, which is evidenced by decreased PD-L1 induction caused by NF-κB inhibitors (Marzec et al., 2008). Notably, the NF-κB inhibitor abolished INF-induced PD-L1 expression, while MAPK, PI3K and STAT3 inhibitors did not. Thus NF-κB also seems to be involved in INF-γ-induced PD-L1 expression (Gowrishankar et al., 2015).

## Glycosylation of PD-L1

*N*-glycosylation is a crucial protein modification that determines protein structure and function, especially the function of membrane proteins. By altering protein conformation, glycosylation may modulate protein activities and protein–protein interactions, such as those between ligands and receptors (Ohtsubo and Marth, 2006). In Western Blot assays, the majority of PD-L1 is detected at 45 kDa representing the glycosylated species, while the non-glycosylated form is detected at 33 kDa. By bioinformatics prediction, mass spectrometry and mutagenesis, PD-L1 was found to be exclusively *N*-glycosylated at N35, N192, N200, and N219 (Li et al., 2016).

**FIGURE 3:**
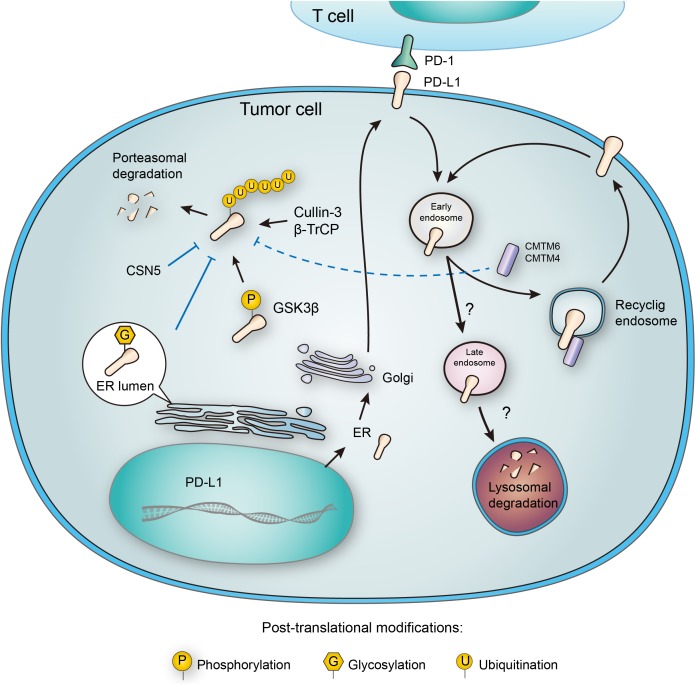
Post-translational modifications and subcellular transportation of PD-L1. As a membrane protein, PD-L1 is extensively modified after its translation. *N*-glycosylation of PD-L1 extracellular domain occurs in the lumen of endoplasmic reticulum (ER), and this modification facilitates the interaction of PD-L1 with lipid membrane. Glycosylation also inhibits phosphorylation by GSK3β, and thereby blocking the ubiquitination by β-TrCP. Deubiquitination by CSN5 also protects PD-L1 from proteasomal degradation. In addition, PD-L1 may also be destructed in lysosome, and this process relies on a series of subcellular transportations from cell membrane to early endosome, late endosome, and finally to lysosome. However, CMTM6 has been found to promote PD-L1 transportation to recycling endosome, causing decreased distribution to late endosome and lysosome. Interestingly, CMTM6 and its homolog CMTM4 may also stabilize PD-L1 by suppressing its ubiquitination.

The PD-L1 molecule containing N192, N200, and N219 residues forms a region that is the prerequisite for PD-L1 binding to GSK3β, and *N*-glycosylation on these sites buries the necessary residues and disrupts the interaction between PD-L1 and GSK3β. Glycogen synthase kinase 3beta (GSK3β), a serine/threonine protein kinase, was originally identified as a regulator of glycogen metabolism (Doble and Woodgett, 2003). When bound to non-glycosylated PD-L1, GSK3β leads to phosphorylation and consequent ubiquitination of PD-L1 (Li et al., 2016) (**Figure 3**). In addition, it was further elucidated that inactivation of GSK3β by activating EGFR enhanced PD-L1 expression by preventing it from being ubiquitinated (Li et al., 2016). Significantly, a small molecular inhibitor of glycosylation, tunicamycin, was found to efficiently decrease PD-L1 expression in cancer cells (Li et al., 2016). Latest results have provided evidence that targeting glycosylated PD-L1 promotes PD-L1 internalization and degradation, leading to eradication of triple-negative breast cancer cells (Li et al., 2018).

## Phosphorylation of PD-L1

Phosphorylation involves in a widespread of regulatory mechanisms in cellular signaling, and may affect the conformation, activity, and interactions of proteins. Although one protein may contain multiple phosphorylation sites, the phosphorylation of PD-L1 has been sparsely reported. As mentioned above in the glycosylation part, GSK3β is a multifunctional switch that mediates the direct phosphorylation of a wide range of substrates, including e IF2B, cyclin D1, c-Jun, c-myc, NFAT, MCl-1, and Snail (McCubrey et al., 2014). It also contributes to the phosphorylation of PD-L1 through an evolutionarily conserved GSK3β phosphorylation motif on PD-L1 (Li et al., 2016) (**Figure 3**). Furthermore, the phosphorylation mediated by GSK3β has been found to initiate the interaction with E3 ligase, which targets proteins to proteasomal degradation (Zhou et al., 2004; Ding et al., 2007; Wang et al., 2018).

Meanwhile, it was reported that treatment of the epidermal growth factor (EGF) would induce tyrosine phosphorylation, together with acetylation and ubiquitination of PD-L1 (Horita et al., 2017). These provide evidential hypothesis for the effects of Gefitinib, an inhibitor of EGFR, in promoting the immune response against breast cancer. Gefitinib was found to cut down on PD-L1 expression and limit its oncogenic potential, therefore promoting T cell immunity. These findings suggest that targeting EGFR by Gefitinib not only suppresses MAPK-dependent tumor proliferation, but also blocks PD-L1-dependent immune suppression (Li et al., 2016). Based on the predicted isoelectric points corresponding to different modifications, the PhosphoSite database has listed potential phosphorylation sites of PD-L1 (basal Isoelectric point = 6.76) (PhosphoSite Plus Protein Page: Pd-L1 Human, 2018). However, no systematic experimental characterization of PD-L1 phosphorylation has been carried out. It also deserves in-depth study how PD-L1 phosphorylation varies and fluctuates in response to distinct microenvironments, therapeutic stresses and interaction with its partner proteins.

## Ubiquitination of PD-L1

Ubiquitination-dependent proteasomal degradation controls the metabolism of many proteins, including membrane proteins like PD-L1 (Zhou et al., 2014). As mentioned above, the EGF treatment may induce tyrosine phosphorylation, acetylation, and ubiquitination of PD-L1 (Horita et al., 2017). The increased PD-L1 mono- and multi-ubiquitination induced by EGF were blocked by gefitinib treatment. Recent study further revealed that ubiquitin E3 is involved in PD-L1 downregulation in EGFR wild-type NSCLC (Wang et al., 2018). In a recent study, cyclin D-CDK4 kinase was reported to destabilize PD-L1 via cullin 3-SPOP, which was proved to be involved in Pd-L1 ubiquitination (Zhang et al., 2018). Surprisingly, the EGF-stimulated PD-L1 mono-ubiquitination not only coexisted with PD-L1 overexpression, but also seemed to occur ahead of its upregulation (Akbay et al., 2013; Chen et al., 2015; Li et al., 2016; Horita et al., 2017). Inhibition of the ubiquitin E1 by blocking its activating enzyme decreased PD-L1 mono- and multi-ubiquitination and total PD-L1 protein expression at the same time, suggesting a possible causal relationship between ubiquitination and overexpression of PD-L1 (Horita et al., 2017).

CMTM6, a type-3 transmembrane protein was recently identified as a positive regulator of PD-L1. Decrease of CMTM6 expression downregulated PD-L1 protein level in a wide range of human tumor cells and in primary human dendritic cells. Apart from CMTM6, its closest family member, CMTM4, was confirmed to share similar function (**Figure 3**). Of note, the enhancement of PD-L1 protein pool stimulated by CMTM6 was not associated with any variation in PD-L1 transcription. Instead, CMTM6 was found to interact with PD-L1 on cell surface, interfering its ubiquitination to prolong its half-life. It was also functionally confirmed that by enhancing PD-L1 protein pool, CMTM6 improves the evasion ability of PD-L1positive tumor cells to immune elimination (Mezzadra et al., 2017).

## Deubiquitination of PD-L1

On the contrary to ubiquitination, deubiquitination of PD-L1 stabilizes the protein from degradation. The deubiquitination and stabilization of PD-L1 significantly affect the inflammatory response or so-called ‘inflammation-mediated anti-tumor immunity’ (Lim et al., 2016). Recently, COP9 signalosome 5 (CSN5) was identified as a crucial protein that promotes the deubiquitination of PD-L1 (Lim et al., 2016) (**Figure 3**). It was reported that tumor necrosis factor alpha (TNF-α), as one of the major inflammatory cytokines secreted by macrophages, plays an important role in maintaining cancer cell evasion from immune system. Mechanistically, TNF-α may activate NF-κB and induce CSN5 expression, leading to PD-L1 stabilization. Consistently, CSN5 has been found to be indispensable for TNF-α-mediated PD-L1 stabilization because of its function in deubiquitinating PD-L1 (Lim et al., 2016). With potential translational significance, the authors found that destabilization of PD-L1 by curcumin, an inhibitor for CSN5, may benefit immunotherapy.

## Subcellular Transportation of PD-L1

PD-L1 functions on the membrane surface, but it may also translocate into the cytoplasm. Many membrane proteins are shuttled between the recycling endosomes and cell surface, and PD-L1 has been tracked in recycling endosomes (Grant and Donaldson, 2009). Furthermore, inhibition of endocytic recycling by primaquine caused vast depletion of membrane PD-L1 protein level in wild-type cells. These results suggest that: first, a large proportion of membrane PD-L1 undergoes metabolism and internalization continuously; second, the dynamic recycling and releasing of PD-L1 maintains the amount of PD-L1 located on cell membrane (Burr et al., 2017). Notably, CMTM6, recognized as a PD-L1 regulator, is predominantly identified in recycling endosomes together with TFRC and RAB11, factors that define the endocytic recycling compartment. What’s more, CMTM6 co-localizes with PD-L1 both on the plasma membrane and in recycling endosomes, so that CMTM6 functions as a protector of PD-L1 that prevents it from being targeted for lysosome-mediated degradation and increases its protein pool (**Figure 3**).

Interestingly, membrane and cytoplasmic PD-L1 expression is more significant in macrophage cells than in cancer cells (Gong et al., 2017). Studies have been done to test PD-L1 molecule in peripheral blood mononuclear cells (PBMC) and surprisingly revealed a novel human PD-L1 splice variant in activated PBMC. Further studies compared the conventional isoform with the novel isoform and found distinct localization patterns between both proteins. Specifically, the conventional isoform is predominantly expressed on the plasma surface, while the novel isoform is distributed mainly on intracellular membrane. The alternative splicing of PD-L1 may be a posttranscriptional regulator that modulates PD-L1 expression as well as its function in determining the outcome of specific immune responses in the peripheral tissues (He et al., 2005).

In addition to its cellular distribution, PD-L1 has also been detected outside the cells, proposing its potential role as a semi-invasive biomarker. An A/C polymorphism at position 8923 was detected together with increased level of plasma soluble PD-L1 (sPD-L1) in NSCLC patients, especially those with adenocarcinoma (Cheng et al., 2015). Investigation is now undergoing to define the value of plasma PD-L1 protein levels as a predictive biomarker of prognosis in NSCLC and also as a reliable companion diagnostics for individualized treatment with ICBT (Zhu and Lang, 2017).

## Lysosomal Degradation of PD-L1

Unlike cytosolic proteins, many membrane proteins are mainly degraded through the lysosomal pathway. As mentioned in the ubiquitination part, CMTM6 reduces PD-L1 ubiquitination and increases its stability (Mezzadra et al., 2017). Interestingly, different opinion presents another explanation about the stabilization of membrane PD-L1 by CMTM6. In addition to its expression at the plasma membrane, CMTM6 is predominantly identified in recycling endosomes (Zhang et al., 2018). Although CMTM6 is not required for PD-L1 maturation, it functions in protecting PD-L1 from lysosome-mediated degradation (Burr et al., 2017). Thus, CMTM6 depletion, via the reduction of PD-L1, significantly alleviates the suppression of tumor-specific T cell activity *in vitro* and *in vivo* (Burr et al., 2017). Although there is no doubt that CMTM6 suppresses PD-L1 degradation, the effect still seems to be indirect, requiring the competitive transportation to the recycling endosome. It remains unclear which protein may directly interact with CMTM6 and transport it to lysosome for degradation (**Figure 3**). Future efforts to clarify this crucial node would benefit the development of alternative PD-L1-targeting approaches.

## Structure-Based Modulation of PD-L1

Some mutations of PD-L1 gene may impede the protein level of PD-1/PD-L1 but others may cause disturbance on protein folding, and therefore disrupt the interaction of PD-1 and PD-L1. PD-1 and PD-L1 bind through the conserved front and side of their Ig variable (Ig V) domains, representing the structural basis for the design of intervention molecules. By locating the loops at the ends of the IgV domains on the same side of the PD-1/PD-L1 complex, a surface is formed, being similar to the antigen-binding surface of antibodies and T-cell receptors (Zak et al., 2017). Several residues have been identified to play important roles in folding and forming the PD-1/PD-L1 interface (Lin et al., 2008). The immune receptor-like loops provide a new surface for further study and potentially the design of molecules that would affect PD-1/PD-L1 binding and thereby regulate the immune system. Multiple peptides and small-molecular compounds have been evaluated in preclinical models, in order to develop novel PD-1/PD-L1 inhibitors (Zak et al., 2017).

In addition to directly block the interaction between PD-1 and PD-L1, methods have also been developed to inhibit the dimerization of PD-L1, and hence the PD-1/PD-L1 interaction. Particularly, this effect could be achieved by small molecular compounds such as BMS-202 and BMS-8, with considerable translational significance (Zak et al., 2017). Since small molecules behold advantages in terms of production scale, quality standardization, pharmacological kinetics and tissue distribution, it is of enormous interest to discover small molecular drugs targeting the PD-L1/PD-1 axis (Lin et al., 2008). Despite the structural insights provided by recent crystallographic research, it is still unclear how the reported PTMs, e.g., glycosylation, phosphorylation, ubiquitination, etc., may affect the conformation and molecular interactions of PD-L1/PD-1. Understanding these detailed processes would also improve the confidence of structure-based drug design targeting this crucial immune suppression signaling pathway.

## Significance of Combined Intervention

PD-L1-targeted ICBT is a promising breakthrough in the field of cancer immunotherapy, but primary and acquired resistances have presented enormous challenges in this fast-evolving area (Pardoll, 2012; Spranger et al., 2016; Zaretsky et al., 2016; Sharma et al., 2017; Zhao and Subramanian, 2017). It has been suggested that the post-treatment positive conversion of PD-L1 expression may be a cause of resistance (Haratake et al., 2017). The regulatory pathways of PD-L1 are of meaningful potential to be translated into therapeutic approaches for tackling the resistance to ICBT (Lee and Tannock, 2010; Tan et al., 2016; Tang et al., 2016; Maj et al., 2017; Shin et al., 2017; Zhao and Subramanian, 2018). The significant PD-L1 overexpression found in multiple cancer types may be an output of interconnected regulatory network, which involves molecular alterations at genetic, epigenetic, transcriptional, translational, post-translational, and structural levels. In fact, several key regulators of PD-L1 have long been established as cancer-related genes, such as JAK2 (Green et al., 2010; Budczies et al., 2016; Ikeda et al., 2016; Clave et al., 2018), PTEN, MAPK, PI3K, HIF-1α, STAT3 (Marzec et al., 2008; Gowrishankar et al., 2015; Chen et al., 2016), TNFα, NF-κB (Gowrishankar et al., 2015), and INF-γ, etc. Existing small molecular compounds targeting these genes/pathways may be repurposed for modulating PD-L1, thus providing readily tools to improve T cell-dependent anticancer immunity. Likewise, the discovery of key post-transcriptional modifications (PTMs) that control PD-L1 stability such as glycosylation, phosphorylation, and ubiquitination also provide alternative strategies for targeting PD-L1 (Zhou et al., 2004; Ding et al., 2007; Li et al., 2016; Lim et al., 2016; Horita et al., 2017). It is worthy to further analyze the function of curcumin (CSN5 inhibitor) and tunicamycin (glycolysis inhibitor) in suppressing PD-1/PD-L1 signaling *in vivo* and in preclinical models. The inhibitors o In addition, the connection between cancer metabolism and resistance to immunotherapy suggests potential benefit for combined targeting of tumor glycolysis and PD-1/PD-L1 axis (Koukourakis et al., 2005; Vaupel and Mayer, 2007; Wilson and Hay, 2011; Shehade et al., 2014; Marchiq and Pouyssegur, 2016; Feng et al., 2017). Apart from controlling the abundance of PD-L1 in cells, the mechanisms underlying PD-L1 transportation and structural modulation may also provide novel strategies to optimize the blockage of PD-L1 (van Weert et al., 2000; He et al., 2005; Lin et al., 2008; Cheng et al., 2015). With the multifaceted regulation of PD-L1 being revealed, it would be more feasible to develop complementary therapies to sustain the response once cancer cells acquire resistance to the initial treatment.

## Outstanding Challenges

The prosperity and challenges of immunotherapies targeting the PD-1/PD-L1 axis warrant increasing attentions by biological and pharmaceutical scientists. In our opinion, several research directions would be especially beneficial to a sustained improvement of ICBT.

Firstly, the regulation of PD-L1 should be further clarified in more specified conditions, considering the variations in tumor regions and developmental stages. It has been suggested that PD-L1 expression may differ considerably on the tumor boundary. Cells located here have higher accessibility where immune cells encounter the tumor cells. Thus, tissue sampling by traditional methods may not robustly capture such alterations and result in low fidelity in different assays such as Western Blot, qPCR and microarray tests. On the other hand, hypoxia-related induction of PD-L1 is more likely to occur in the center of solid tumors where oxygen is less accessible. Moreover, our recent study found that PD-L1 is significantly upregulated in metastatic CRCs while compared to primary tumors (Wang H.B. et al., 2017). Thus, the regulation of PD-L1 during metastasis and its corresponding biomarker significance should be considered differentially from those in the primary tumors. To investigate the regulation of PD-L1 in tumors, it is essential to precisely mark the region and stage (e.g., primary vs. metastatic, pre-treatment vs. post-treatment, etc.) of a particular patient, because these variations are associated with the indicated mechanisms.

Secondly, the link between PD-L1 expression and cancer subtyping has been investigated based on genomic and transcriptomic characterizations of tumors. In many tumors, the microsatellite instability (MSI) subtype is linked to PD-L1 positivity and considered as a key factor indicating the suitability for checkpoint blockade therapy (Xiao and Freeman, 2015; Dudley et al., 2016). Even though, more comprehensive understanding on the implications of PD-L1 in cancer subtyping should also be founded by insights into the epigenetic and metabolic reprograming of cancer cells. As described previously, epigenetic and metabolic alterations in tumors are emerging as crucial factors affecting the abundance of PD-L1. In a translational perspective, significant and functional alterations at these facets may also present novel biomarkers and intervention opportunities.

Thirdly, it deserves tremendous efforts to clarify the overlaps and differences between PD-L1 and its homolog PD-L2 in their functions and regulations in various tumors. Although PD-L2 was initially considered to be mainly expressed in immune cells, recent studies have revealed its positive expression in different tumor cells with potential prognostic significance. As an example, we found that PD-L2 is expressed in a considerable subset of CRC cells, with independent association with poor patient survival (Wang H. et al., 2017). It is thus of interest to clarify the relative importance of PD-L1 and PD-L2 in a specific tumor type. Will one protein compensate the function of the other, or be upregulated when its homolog is blocked in immunotherapy? Which ligand of PD-1 may play a predominant role in suppressing T-cell immunity in a given cancer type of patient, and should this be considered when optimizing the strategy for immunotherapy? These questions should be addressed, in order to understand and improve the effectiveness and sustainability of ICBT.

Finally, the structure-based drug design targeting PD-L1 may not be limited in the binding surface to PD-1 or the site mediating its dimerization. If allosteric control of PD-L1 activity could be identified, additional approaches targeting PD-L1 would be feasible. Moreover, the protein interactions between PD-L1 and its reported regulators (e.g., CSN5, CMTM6, etc.) could be characterized in and enough resolution, rational design of blocking peptides or compounds may also be developed. In other words, basic research about the structural dynamics and detailed interaction sites of PD-L1 may provide additional resources for the development of *de novo* PD-L1 targeting approaches.

## Conclusion

Immune checkpoint blockade therapy represents a breakthrough in cancer treatment, but the primary and acquired resistance to immunotherapy warrant further efforts to understand the multifaceted regulation of PD-L1 in cancer. As a cell surface protein that responds to microenvironment stimuli, PD-L1 reacts promptly to balance the outside stresses and inside requirements of cells, representing a key node in the cancer signaling network. In this scenario, the effective and sustained targeting of PD-L1 has to take the complexity of its regulation into account. Identification of the exact causes of PD-L1 upregulation and responsive functional compensations in a broader range of molecular events would improve the targeting specificity and efficiency. A chasm is yet to be crossed by obtaining small molecular inhibitors of PD-L1 in addition to antibody drugs, to improve the cancer distribution and metabolic kinetics of immunotherapeutic medicines. Current approaches for targeting PD-L1 could also affect its normal functions in immune cells, with expected unwanted effects. In these scenarios, targeting PD-L1 effectively and specifically in cancer cells remains a Gordian knot.

## Author Contributions

YW and JX wrote the manuscript. HW, HY, CL, and J-YF contributed to revisions of the manuscript.

## Conflict of Interest Statement

The authors declare that the research was conducted in the absence of any commercial or financial relationships that could be construed as a potential conflict of interest.
